# Cognitive Testing in People at Increased Risk of Dementia Using a Smartphone App: The iVitality Proof-of-Principle Study

**DOI:** 10.2196/mhealth.6939

**Published:** 2017-05-25

**Authors:** Susan Jongstra, Liselotte Willemijn Wijsman, Ricardo Cachucho, Marieke Peternella Hoevenaar-Blom, Simon Pieter Mooijaart, Edo Richard

**Affiliations:** ^1^ Academic Medical Center Department of Neurology University of Amsterdam Amsterdam Netherlands; ^2^ Leiden University Medical Center Department of gerontology & geriatrics University of Leiden Leiden Netherlands; ^3^ Leiden Institute of Advanced Computer Sciences LIACS University of Leiden Leiden Netherlands; ^4^ Institute of Evidence-based Medicine in Old Age - IEMO, LUMC Leiden Netherlands; ^5^ Donders Institute for Brain, Behaviour and Cognition Department of Neurology Radboud UMC Nijmegen Netherlands

**Keywords:** telemedicine, cognition, neuropsychological tests

## Abstract

**Background:**

Smartphone-assisted technologies potentially provide the opportunity for large-scale, long-term, repeated monitoring of cognitive functioning at home.

**Objective:**

The aim of this proof-of-principle study was to evaluate the feasibility and validity of performing cognitive tests in people at increased risk of dementia using smartphone-based technology during a 6 months follow-up period.

**Methods:**

We used the smartphone-based app iVitality to evaluate five cognitive tests based on conventional neuropsychological tests (Memory-Word, Trail Making, Stroop, Reaction Time, and Letter-N-Back) in healthy adults. Feasibility was tested by studying adherence of all participants to perform smartphone-based cognitive tests. Validity was studied by assessing the correlation between conventional neuropsychological tests and smartphone-based cognitive tests and by studying the effect of repeated testing.

**Results:**

We included 151 participants (mean age in years=57.3, standard deviation=5.3). Mean adherence to assigned smartphone tests during 6 months was 60% (SD 24.7). There was moderate correlation between the firstly made smartphone-based test and the conventional test for the Stroop test and the Trail Making test with Spearman ρ=.3-.5 (*P*<.001). Correlation increased for both tests when comparing the conventional test with the mean score of all attempts a participant had made, with the highest correlation for Stroop panel 3 (ρ=.62, *P*<.001). Performance on the Stroop and the Trail Making tests improved over time suggesting a learning effect, but the scores on the Letter-N-back, the Memory-Word, and the Reaction Time tests remained stable.

**Conclusions:**

Repeated smartphone-assisted cognitive testing is feasible with reasonable adherence and moderate relative validity for the Stroop and the Trail Making tests compared with conventional neuropsychological tests. Smartphone-based cognitive testing seems promising for large-scale data-collection in population studies.

## Introduction

The global prevalence of dementia is likely to increase in the coming years, mainly due to the growing population with an increased life expectancy [1]. To investigate interventions to prevent dementia, large sample sizes with long follow-up are required [2]. Assessment of cognitive functioning over time is important for early detection of cognitive decline in longitudinal dementia prevention studies. Conventional neuropsychological examination is burdensome, time-consuming, and expensive and therefore hardly feasible in large-scale studies with long follow-up. To get informed about cognitive functioning without the need for full neuropsychological examination, innovative solutions are required.

New technology is rapidly adopted by older generations, illustrated by a steady increase in the Internet and smartphone use over the last years [3]. Using smartphone technology, remote monitoring of health parameters such as physical activity and blood pressure have already been widely studied and found feasible, also in older populations [4,5]. Smartphones are likely to be the principal platform for the development of the next generation of clinical care and research [6]. Smartphone-assisted cognitive testing would provide the ability to assess cognitive functioning rapidly and repeatedly in a noninvasive manner, at a convenient moment, and without generating high costs. Experience with smartphone use during a clinical cognitive assessment has already been tested [7], paving the way to integration in a home setting. Feasibility and validity of smartphone-based cognitive testing has been described, although narrowed down to specific patient groups or a specific cognitive test [8-11]. Despite these advances made in conducting smartphone research, little is known in terms of the feasibility and validity of applying multiple cognitive tests using smartphone-based technologies for clinical research in larger populations. Implementation of an app is only feasible if participants are compliant [12] and the technical performance is optimal [13].

The aim of this study was to investigate the feasibility and validity of a cognitive test battery using smartphone-assisted technology in healthy adults, during a 6 months follow-up period.

## Methods

### Study Participants

We recruited participants at increased risk of cognitive decline and dementia, operationalized as a parental history of dementia [14]. These persons are highly motivated to participate in a monitoring study to support preventive strategies for dementia, and therefore suitable for a proof-of-principle study [15].

Participants were included if: (1) they were 50 years or older, (2) at least one of their parents was diagnosed with any form of dementia, (3) they knew how to handle and were in possession of a smartphone with iOS or Android (version 2.3.3 or higher) software, (4) they had no dementia or any other cognitive disorder, and (5) they had no medical history of stroke or transient ischemic attack.

Participants were recruited through advertisements at memory outpatient clinics, nursing homes, general practices, and using the communication channels (website and newsletter) of the Dutch Alzheimer Foundation. People were asked to contact the study center and if all of the inclusion criteria were met, participants received detailed study information in print and an appointment for baseline measurement was made. Enrolment and follow-up took place from September 2013 to January 2015. Written informed consent was obtained from all participants at the baseline study visit. The study was approved by the medical ethical committee of Leiden University Medical Center (LUMC), the Netherlands.

### iVitality Platform

iVitality is a Web-based research platform that consists of a website, a smartphone-based app, and sensors that are connected with or already integrated in the smartphone to measure health characteristics including cognitive function, blood pressure [4], physical activity (integrated pedometer), and life style (with questions about health and mood). The smartphone-based app was installed during the baseline assessment and the sensors were activated if participants were officially included in the study, until the end of follow-up. Participants could log on to the website to overlook the measurements and results of their performance on the app. Participants received alerts from the iVitality smartphone app to perform a test or measurement (eg, cognitive test or blood pressure) on their smartphone.

### Study Design

Participants visited the study center at LUMC or Academic Medical Center (AMC) at baseline, where they received information about the study and the smartphone-based app was installed and explained. During this visit, baseline measurements were performed by a study physician or research nurse. Afterwards, during a 6 month follow-up period, participants received messages on their smartphone, reminding them to voluntarily perform a specific cognitive test (Table 1). Alert moments were chosen in a way that every test had at least four reminder moments evenly spread during the 6 month follow-up period. Table 1 indicates on what day since baseline the message was sent for every test to every individual participant. The smartphone app collected data from the tests and provided feedback to the participant by showing the results of their measurements. A secured Internet connection transferred the data to the website and the database of the study center.

**Table 1:** Message moment per cognitive test during follow-up.

Weeks in study	1	3	5	7	9	11	13	15	17	19	21	23	25
Memory-Word	Day 1		Day 29					Day 99					Day 169
Trail Making test	Day 2			Day 43					Day113				Day 170
Stroop	Day 3				Day57					Day127			Day 171
Reaction Time test	Day 4					Day71					Day 141		Day 172
Letter-N-Back		Day 15					Day 85					Day 155	Day 173

### Baseline Measurements

In preparation for the first visit to the study center, all participants completed a Web-based questionnaire including questions about level of education, medical history, and medication use. The study physician measured parameters including weight, height, and blood pressure of all participants.

Cognitive function at baseline was tested using five neuropsychological tests to assess global cognitive function, executive function, attention, and immediate and delayed recall. The mini-mental state examination (MMSE) [16] was used to evaluate global cognitive function. The 15-Word Verbal Learning test (15-WVLT) [17] was used to assess immediate and delayed recall. The Trail Making test (TMT) [18], parts A and B, were used to measure attention and executive function. The Stroop-Color-Word test [19] was used to test selective attention.

### Smartphone-Based Cognitive Tests

Five digital versions of cognitive tests were developed for the iVitality smartphone app based on existing neuropsychological tests, but carefully adapted for smartphone use.

The Memory-Word test was based on the 15-WVLT [17]. A series of 10 words with a fixed time pace was presented to the participants, which they were instructed to remember. Directly afterwards, participants were displayed a list of 20 words, including the 10 words which were presented before, mixed with 10 new words. Participants had to press “yes” or “no” for recognition. Each correct and incorrect response was recorded.

The TMT, based on the original TMT part A and B [18], consisted of four parts of increasing complexity in which participants had to make a trail connecting 12 circles. In part 1, the circles contained numbers in ascending order (1-2-3), part 2 contained letters in ascending order (A-B-C), part 3 contained numbers and letters alternating in ascending order (1-A-2-B), and in part 4, numbers and letters had to be connected alternating and in opposing order: numbers ascending, letters descending (1-Z-2-Y). The total time for each part was recorded. This last part was added to decrease the ceiling effect in a cognitively healthy population.

The Stroop color-word test was based on the original Stroop test [19]. In the smartphone version, 30 items were presented in all three parts. Names of colors in black letters (part I), colored blocks (part II) or names of colors in other colored letters (part III) were presented together with multiple-choice answers. Total time to complete each part was recorded.

The Reaction Time test consisted of two parts: in part 1, participants were requested to touch the screen of the smartphone as soon as a presented green box turned blue. In part 2, the green box was again presented, but turned into either a blue or red box. The participants had to touch the screen as soon as possible, only if the blue box appeared. At one random instance an enlarged blue box was presented, as a measure of the startle time. In all parts, the time was recorded between the box turning blue and the moment the participant touched the screen in milliseconds. The time between presenting the enlarged blue box and pressing the screen was recorded as the startle (reaction) time in milliseconds.

The Letter-N-Back test, based on the original N-back test [20], consisted of four parts. A series of letters on the screen of the smartphone was presented in a sequential order. In part 1 (0-back), participants had to touch the screen when the letter “X” appeared (in total 11 items presented); for part 2 (1-back), participants had to touch the screen when the letter that was displayed, was the same as the previous one (in total 11 items presented); in part 3 (2-back), participants had to touch the screen when a letter that was displayed was the same as the one before the previous one (in total 15 items presented); and in part 4 (3-back), they had to touch the screen when the letter that was displayed was the same as the one that was presented before the previous 2 letters (in total 20 items presented). Each correct and incorrect response was recorded.

Prior to each test, a short explanation was displayed. Screenshots of the tests are shown in Multimedia Appendix 1.

### Statistical Analyses

Characteristics of the study participants are reported as mean (SD) for continuous variables and as number (%) for categorical variables.

Feasibility was evaluated by the technical performance of the app and adherence to perform cognitive tests on a smartphone. Validity was studied by assessing the correlation between conventional and smartphone cognitive tests, and the effect on performance of repeated cognitive tests on a smartphone.

For each participant and each test, we assessed adherence during follow-up. Adherence was defined as the actual performance of cognitive test measurements within 1 week of the reminder received through the smartphone app. The technical performance was defined as the ability to function as developed on every participant’s smartphone.

To assess the relative validity of the first performed smartphone test compared with the conventional Stroop and TMT, we calculated the correlation coefficient. Since the test results were generally not normally distributed, we used Spearman correlation coefficient. To investigate systematic differences between conventional and smartphone cognitive tests, we computed z-scores for both and visualized the values in a Bland-Altman plot.

In a sensitivity analysis, we assessed the correlation between the score on the conventional test at baseline and the mean score of all attempts a participant had made on a specific smartphone-based test, to account for (technical) difficulties in the first attempt and a learning curve. In a second sensitivity analysis, we assessed the correlation between the conventional Stroop test and the first smartphone attempt without many mistakes. The participant needed to score at least half of the answers correct, and if not, the following score (of the next attempt) was taken. Since no conventional version of the Letter-N-Back test and the Reaction Time test were done at baseline, we could not assess the relative validity for these tests.

To assess potential learning effects after repeated testing, performance over time on the smartphone cognitive tests were visualized graphically. We analyzed the linear trend in test performance with each attempt using a linear mixed effects model with a random intercept and random slope for attempt within each subject (MIXED procedure). To investigate selective dropout, we performed an additional analysis on the effect of repeated testing including only those participants who performed 9 tests or more.

All analyses were performed using IBM SPSS software (version 23).

## Results

### Baseline Characteristics

**Figure 1:**
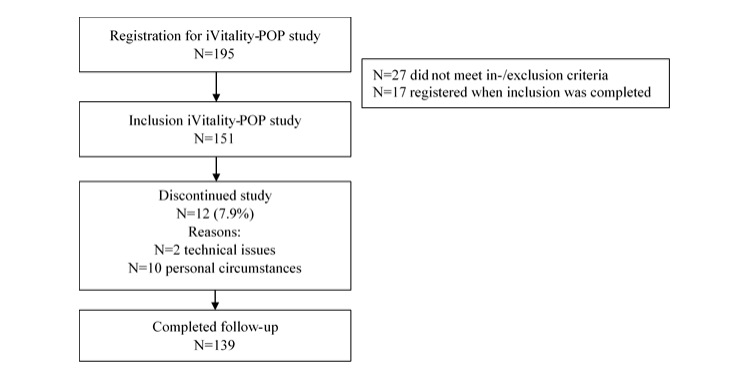
Flowchart inclusion of study participants.

The flowchart for inclusion of participants is shown in Figure 1. The study population consisted of 151 participants. Two participants discontinued the study immediately after baseline visit because of technical issues with their smartphone, so they do not have smartphone measurements. During the follow-up period of 6 months, 12 participants (8.1%, 12/149) discontinued the study.

Baseline characteristics are shown in Table 2. Mean age was 57.3 (SD 5.3) years and 70.9% (107/151) were female. The most commonly used smartphone types were iPhone and Samsung. Around 58.3% (88/151) of the participants had a high education level. None of the participants had an MMSE score below 27 points. More details about the other baseline characteristics are published elsewhere [4,15].

**Table 2:** Baseline characteristics of study participants.

Demographics		Study participants (N=151)
Age (years), mean (SD)		57.3 (5.3)
Female, n (%)		107 (70.9)
**Highest education level^a^, n (%)**			
	Low (<7 years)	16 (10.6)
	Middle (7-12 years)	44 (29.1)
	High (>12 years)	88 (58.3)
Body mass index (kg/m^2^), mean (SD)		26.4 (4.0)
Systolic blood pressure (mmHg), mean (SD)		138 (18.2)
Diastolic blood pressure (mmHg), mean (SD)		85 (10.8)
MMSE^b^, median (interquartile range)		29 (29-30)

^a^Missing data for n=3 participants.

^b^MMSE: mini mental state examination.

### Adherence

**Figure 2:**
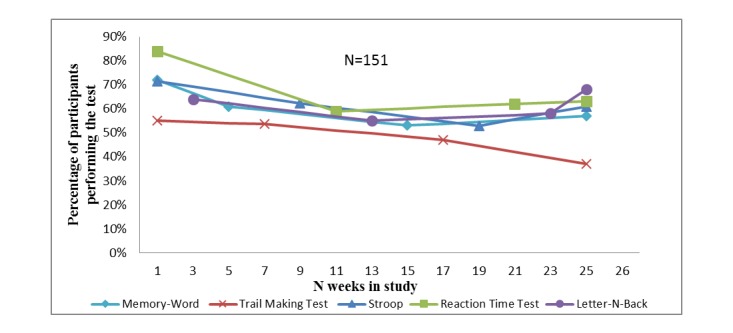
Adherence to smartphone-based cognitive tests.

Adherence to the test program of the five smartphone-based cognitive tests during a 6 month follow-up is shown in Figure 2. Adherence was highest for the Reaction Time test (67%) and slightly lower for the other tests (62% for the Stroop test, 61% for the Memory-Word test, 61% for the Letter-N-Back test, and 48% for the Trail Making test). During the 6 month follow-up, adherence slightly decreased for all tests. Mean adherence per participant was 60% (SD 24.7). When investigating the data for the percentage of participants (calculated from total N=151) who made a test at least once during follow-up, irrespective of timing relative to the reminder, this was 98% for the Reaction Time test, 97% for the Stroop test, 95% for the N-back test, 94% for the Memory-Word test, and 89% for the TMT.

### Relative Validity of the Smartphone Test Compared With the Conventional Test

Raw test scores of the conventional tests at baseline and the firstly performed smartphone tests are described in Multimedia Appendix 2. Since the smartphone-based tests were based on the conventional tests but not identical, direct comparison between the raw test scores is not possible using absolute values.

**Table 3:** Relative validity of conventional cognitive test at baseline compared with cognitive test on smartphone.

Test	n	Conventional versus first performed smartphone test		Conventional versus mean score of all performed smartphone tests	
		ρ CC^a^	*P* value	ρ CC	*P* value
Stroop panel 1	146	.36	<.001^b^	.42	<.001^b^
Stroop panel 2	146	.31	<.001^b^	.36	<.001^b^
Stroop panel 3	146	.50	<.001^b^	.62	<.001^b^
TMT^c^ numeric	135	.38	<.001^b^	.48	<.001^b^
TMT alphanumeric	135	.39	<.001^b^	.43	<.001^b^

^a^CC: correlation coefficient.

^b^Significant at the <.001 level.

^c^TMT: Trail Making test.

The association between the conventional cognitive test made at baseline and the corresponding firstly performed cognitive test on the smartphone is shown in Table 3. There was moderate correlation between the smartphone-based test and the conventional test for the Stroop test (panel 3) and the TMT with ρ=.5 and .4 respectively.

The sensitivity analysis in which we investigated the correlation between the conventional test and the mean score of all performed corresponding smartphone tests during follow-up showed higher correlation coefficients for both tests compared with the correlation with the first performed cognitive test (Table 3).

The number of mistakes made by the participants in the conventional Stroop test was very low and randomly distributed, and therefore not accounted for in the analysis. The number of mistakes in the smartphone-based Stroop test was accounted for in a sensitivity analysis (Multimedia Appendix 3). This showed a higher correlation coefficient for all three panels compared with the correlation with the first performed cognitive test when not accounted for mistakes (panel 1: ρ=.39, panel 2: ρ=.33, and panel 3: ρ=.57).

**Figure 3:**
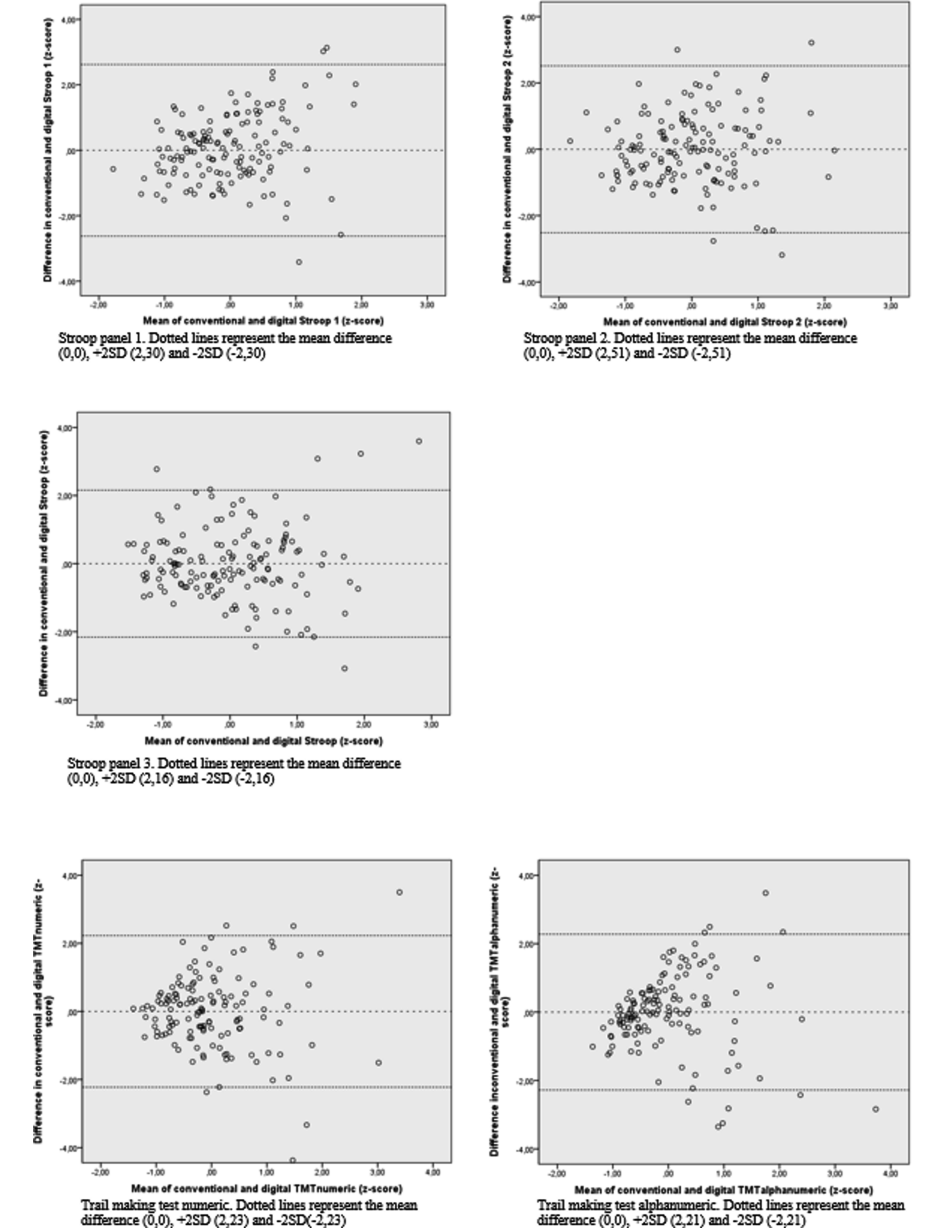
Systematic differences between conventional and smartphone-based cognitive tests in a Bland-Altman-plot. All values are standardized in z-scores.

The Bland-Altman plots of tests which showed moderate correlation (Figure 3) show that the difference between the measurements was randomly distributed over the mean of the measurements. However, inspection of the Bland-Altman plot suggests that for the TMT (numeric and alphanumeric), correlation decreases with increasing time needed to complete the test.

### Repeated Cognitive Testing

**Figure 4:**
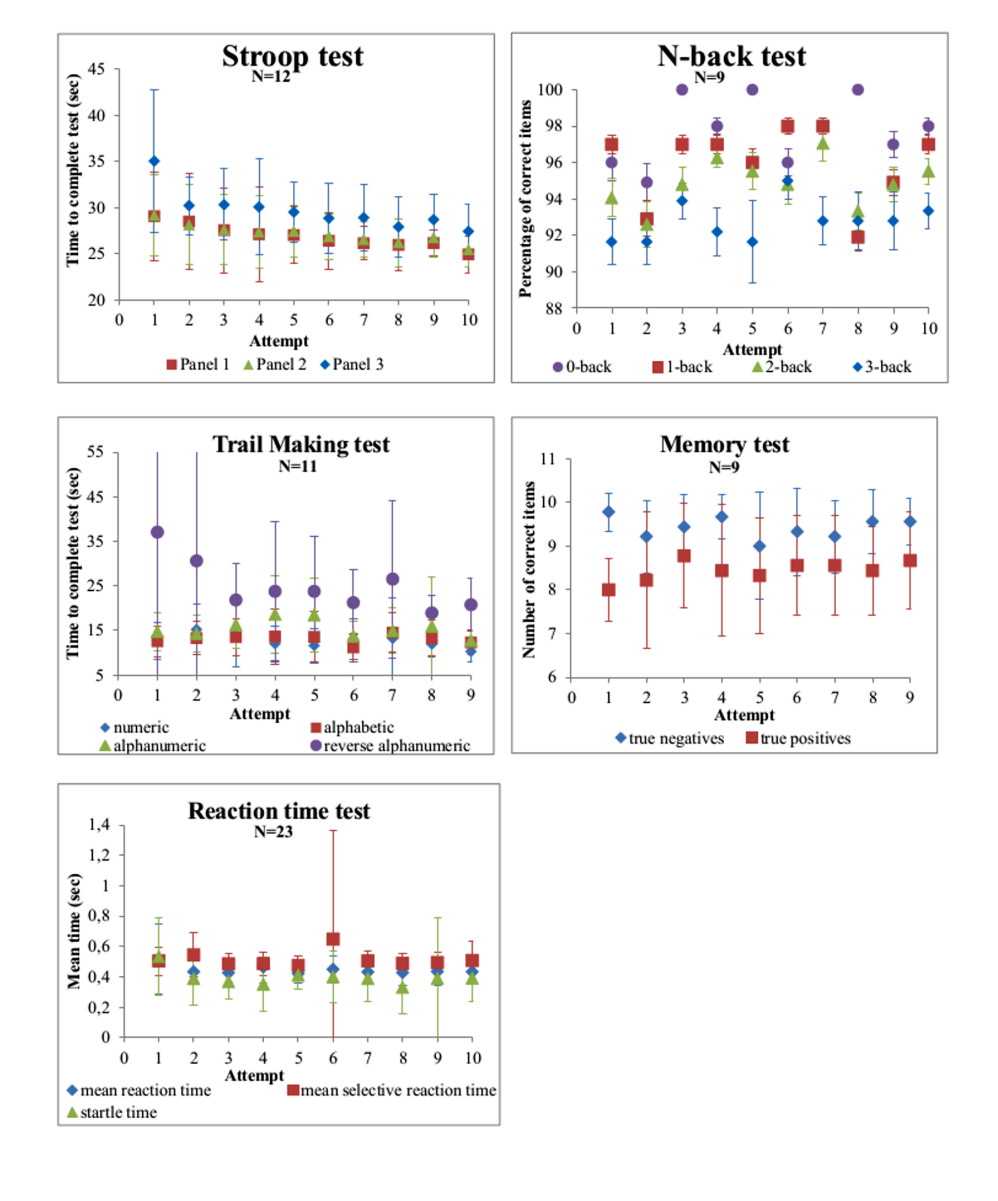
Effect of repeated cognitive tests on smartphone.

The trend in test scores for each smartphone-based test is shown in Figure 4. With increasing number of test repeats, the number of participants contributing to the data decreased since each test was actively offered 4 times during the study, so any excess number of performed tests is on participants’ initiative. The performance on the Stroop test improved for each panel with almost 1 sec per attempt (panel 3: beta=−.93, *P*<.001) and the reversed alphanumeric TMT improved with 1.8 sec per attempt (beta=−1.81, *P*<.001). The performance on the N-back, the Memory-Word, and the Reaction Time test remained virtually stable over time.

The sensitivity analysis in participants who performed the tests at least nine times showed similar results (Multimedia Appendix 4).

## Discussion

### Principal Findings

Our study shows that smartphone-based cognitive testing in cognitively healthy adults over 50 years of age is feasible and that motivated research participants are reasonably adherent to regular testing following an alert on their smartphone. Of the cognitive tests developed in iVitality, the smartphone-based Stroop test and the TMT have a moderate correlation with conventional tests. Repeated testing leads to improved test scores with increasing number of tests performed, suggesting a learning effect for the Stroop test and the TMT.

Adherence to smartphone tests in trial setting varies between studies (17%-90%) [21-23]. These mixed percentages can be explained by the broad definition of adherence in smartphone interventions considering different frequencies, lengths, and intensities of use. Adherence of our participants is relatively good (60%) compared with these studies. The high frequency of reminders the participants received not only for the cognitive tests, but also for the other measurements in the iVitality POP study, could have caused a certain degree of alarm fatigue. This could have reduced the adherence and might explain the variability in adherence in our study. Participants were most adherent to the Reaction Time test. Potential reasons are that the test is easy, not very time-consuming, and does not require processing of information. Only 2 participants (1.3%) could not perform the smartphone tests because of technical problems. This suggests that repeated smartphone-based neuropsychological testing outside the context of a research center is also technically feasible.

Few studies have been performed to validate cognitive testing using a smartphone, usually in the context of a specific disorder or healthy young people [8,10,22]. The moderate correlations in our study for the attention and executive function tasks are comparable with correlations found in a other study investigating cognitive smartphone apps focusing on working memory and perceptual speed [24]. Another Stroop smartphone app was already validated to diagnose covert hepatic encephalopathy [9], but was not compared with the conventional Stroop test [19]. The moderate association found between the conventional Stroop test and a smartphone Stroop test was not found before [22]. This is also the first study investigating the TMT on a smartphone compared with the conventional version [25] with a moderate correlation.

The correlation coefficient increases for all smartphone-based tests with more attempts and when leaving the scores out from participants who made many mistakes in the smartphone Stroop test (Multimedia Appendix 3), implicating that technical challenges while performing the test may have to be overcome. Our participants received short digital instructions prior to the smartphone-based tests in an attempt to limit the influence of technical issues. Nevertheless, the first attempt could be less reliable because of misunderstanding. The mean performance reduced random measurement error and therefore resulted in stronger associations. Especially for the Stroop test we noticed that some participants made a lot of mistakes in the first attempt (more than half of the answers were incorrect), indicating misunderstanding and implicating the need for more explanation on beforehand in further research.

In line with our findings, another study that also developed a Letter-N-Back test and Reaction Speed test for the smartphone did not observe a learning effect over time [22]. The fact that we did not find an improvement on performance in the Memory-Word, Letter-N-Back, and Reaction Speed tests can be due to the ceiling effect in our sample of participants without any cognitive complaints.

### Limitations

This proof-of-principle study has several limitations. We selected participants with a parental history of dementia and therefore they are highly motivated to participate. This may have introduced a selection bias toward better adherence, which reduces the external validity. Another limitation is that we could not validate every smartphone test to a conventional test administered at baseline. Future studies must try to develop a more comparable smartphone test. Strengths of this study are the relatively large sample size for a proof-of-principle study, the moderate level of adherence, and the validation of part of the tests to conventional neuropsychological tests.

### Conclusions

Taken together, the results of this proof-of-principle study show that smartphone cognitive testing in healthy older individuals is feasible and yields valid test results. It allows for repeated testing to observe changes over time while reducing the need for face-to-face contact, making it time-efficient, less burdensome for research participants, and less expensive. The tests should be considered as screening tests to detect changes over time, rather than replacing conventional neuropsychological test batteries. It may be particularly useful for large-scale data-collection in population studies with long follow-up requiring repeated testing.

Before implementation of this type of tests, further research should focus on criterion validity to investigate whether the tests adequately pick up cognitive decline both cross-sectionally as well as longitudinally. To reduce a potential learning effect, alternative versions of the tests could be developed, although for longitudinal research this is less important since the learning effect seemed to wane in our study.

## Appendix

Multimedia Appendix 1 Screenshots of cognitive tests on smartphone.

Multimedia Appendix 2 Mean test results of conventional cognitive tests at baseline and first made cognitive test on a smartphone.

Multimedia Appendix 3 Relative validity of the first performed cognitive test without many mistakes compared to the conventional cognitive test at baseline.

Multimedia Appendix 4 Effect over time for repeated cognitive tests (only for the participants who made the test at least 9 times).

## Abbreviations

AMC: Academic Medical Center
LUMC: Leiden University Medical Center
MMSE: mini-mental state examination
SD: standard deviation
TMT: Trail Making test
15-WVLT: 15-Word Verbal Learning test
CC: correlation coefficient
