# Supplementary figures and images for: Cognitive Testing in People at Increased Risk of Dementia Using a Smartphone App: The iVitality Proof-of-Principle Study
Source: JMIR Mhealth Uhealth. 2017 May 25;5(5):e68. doi: 10.2196/mhealth.6939 (PMC5465383; doi:10.2196/mhealth.6939)

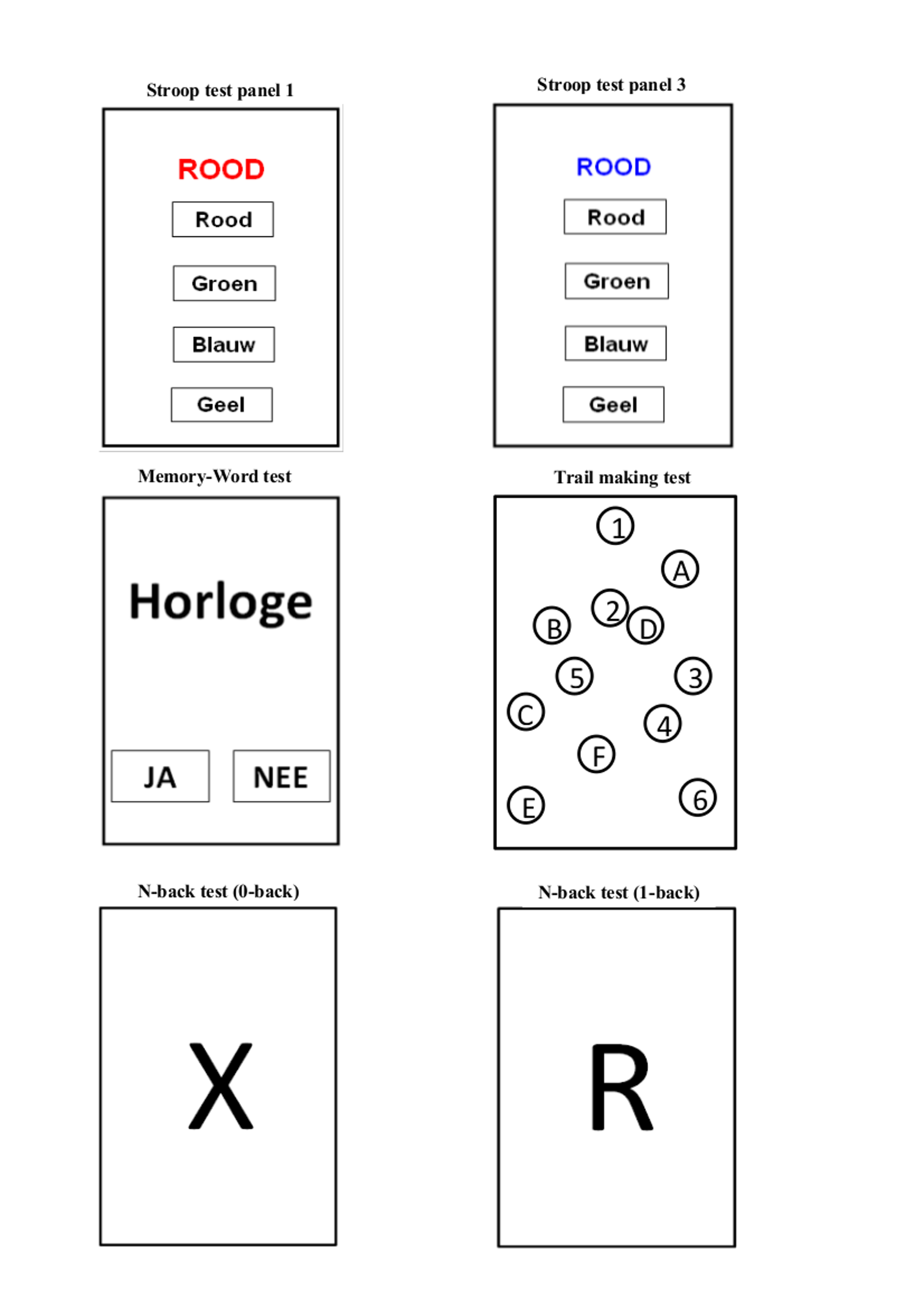

Supplement: Multimedia Appendix 1 [file mhealth_v5i5e68_app1.png]

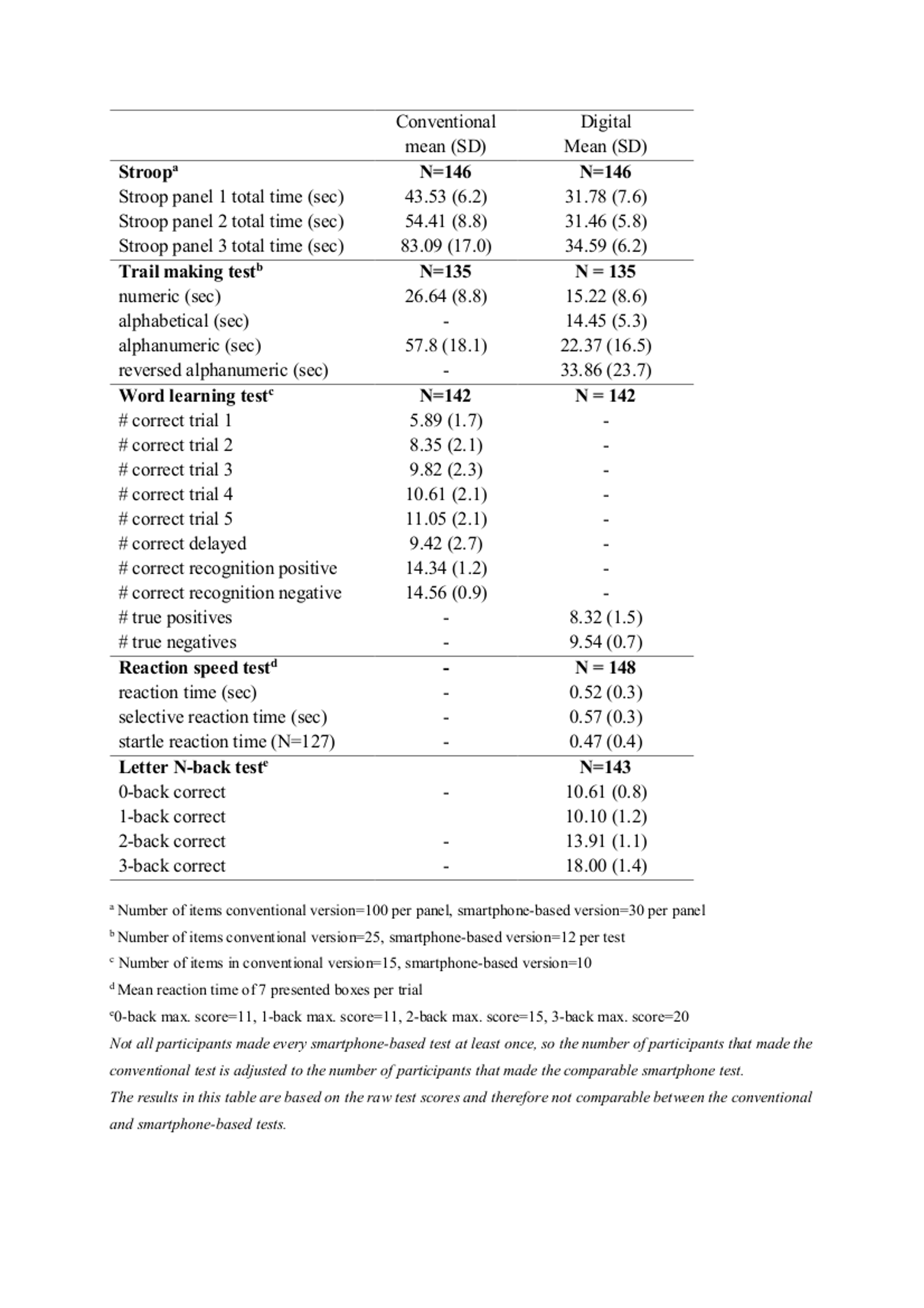

Supplement: Multimedia Appendix 2 [file mhealth_v5i5e68_app2.png]

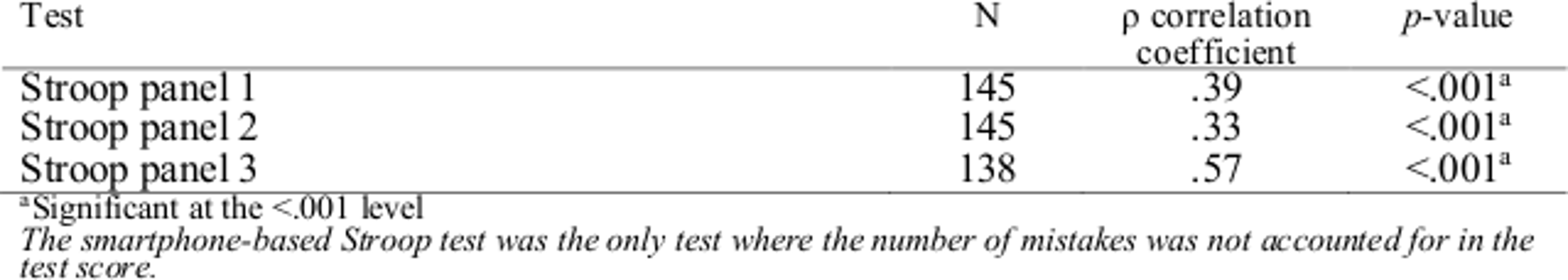

Supplement: Multimedia Appendix 3 [file mhealth_v5i5e68_app3.png]

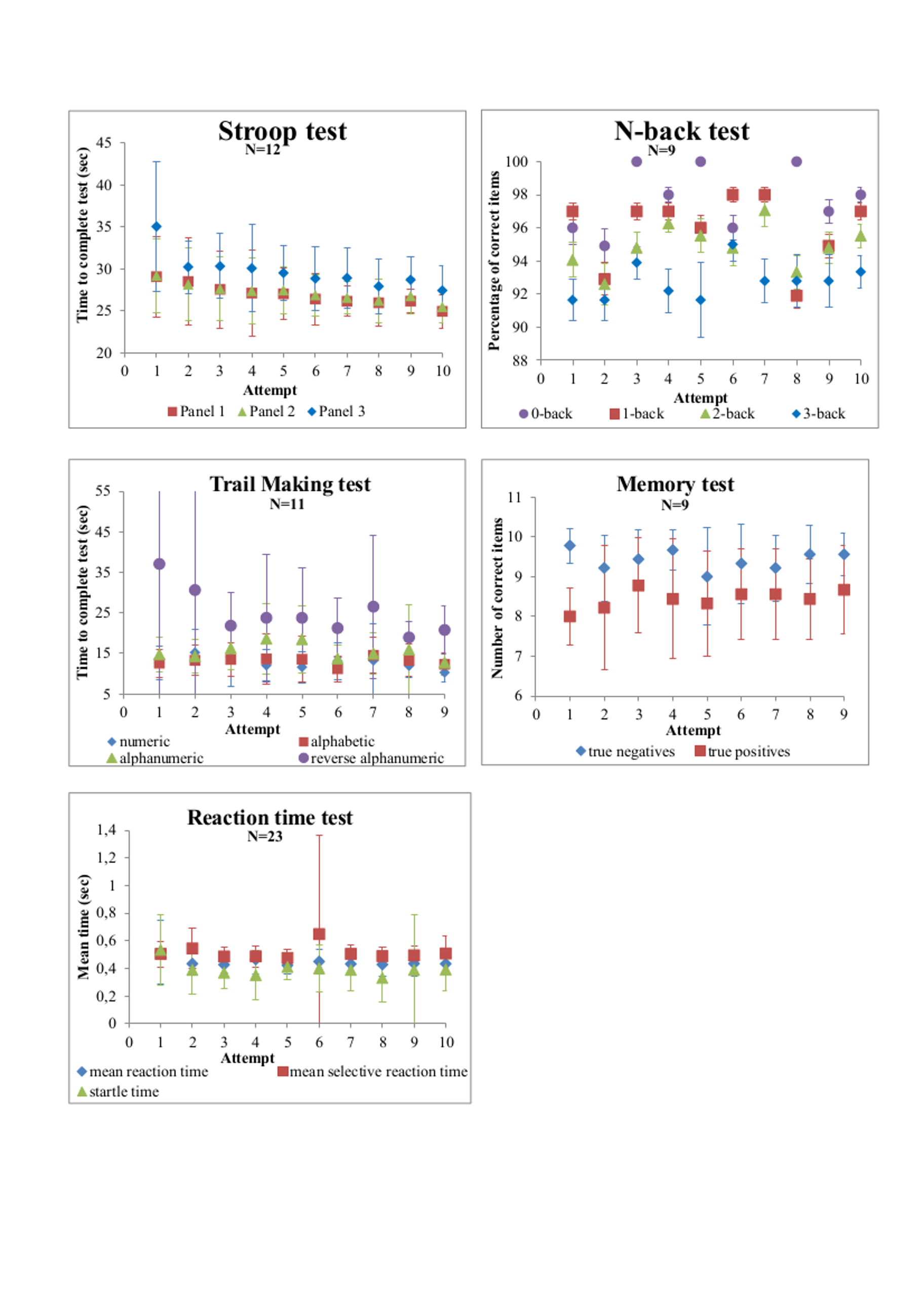

Supplement: Multimedia Appendix 4 [file mhealth_v5i5e68_app4.png]
